# HER2-Selective and Reversible Tyrosine Kinase Inhibitor Tucatinib Potentiates the Activity of T-DM1 in Preclinical Models of HER2-positive Breast Cancer

**DOI:** 10.1158/2767-9764.CRC-23-0302

**Published:** 2023-09-25

**Authors:** Devra Olson, Janelle Taylor, Kelsi Willis, Kelly Hensley, Sean Allred, Margo Zaval, Lauren Farr, Robert Thurman, Nishi Jain, Renee Hein, Michelle Ulrich, Scott Peterson, Anita Kulukian

**Affiliations:** 1 Seagen Inc., Bothell, Washington.

## Abstract

The oncogenic receptor HER2 is overexpressed in many cancers, including up to 20% of breast cancers. Despite the availability of HER2-targeted treatments, patients’ disease often progresses during therapy, underscoring the need for novel treatment strategies. The addition of tucatinib, a reversible, highly selective HER2 tyrosine kinase inhibitor (TKI), to treatment with trastuzumab and capecitabine significantly improved survival outcomes of patients with HER2-positive metastatic breast cancer, including those with active brain metastases. We rationalized that combining tucatinib with other HER2-targeting agents with complementary mechanisms of action would further increase efficacy against tumors. We characterized the activity of tucatinib with the antibody–drug conjugate T-DM1 in preclinical models of breast cancer, including HER2-positive breast cancer cells and patient-derived xenograft (PDX) models. Mechanistic details on tucatinib activity were obtained in internalization and catabolism studies. In combination, tucatinib and T-DM1 showed an enhanced, often synergistic, cytotoxic response and demonstrated improved antitumor activity *in vivo*, including in PDX models refractory to T-DM1 single-agent activity. Mechanistically, tucatinib mediated an increase in inactive HER2 molecules at the cell surface through inhibition of HER2 ubiquitination, resulting in increased internalization and catabolism of T-DM1. The combination was correlated with enhanced HER2 pathway inhibition, decreased proliferation, and increased apoptosis. In a xenograft model of brain metastasis, tucatinib penetrated intracranial tumor tissues, inhibiting tumor growth and improving survival. These results suggest that tucatinib may be the optimal TKI partner for HER2-targeted therapies and support clinical studies of its combination with T-DM1, including in patients with brain metastases.

**Significance:**

The preclinical findings in breast cancer models presented here demonstrate that combining tucatinib with T-DM1 enhances the antitumor activity of either agent alone, supporting clinical studies of the combination in HER2-positive breast cancer, including in patients with brain metastases, which remains an important unmet medical need.

## Introduction

From the time of the initial characterization of *HER2* as an oncogenic receptor tyrosine kinase and a critical driver of HER2-positive disease, HER2 overexpression has been linked to several cancers including those affecting colorectal, gastric, lung, ovarian, and salivary gland tissues, as well as 15%–20% of breast cancers (1–5). While historically, HER2-positive breast cancer had a worse prognosis than other breast cancer subtypes (6, 7), the introduction of HER2-targeted therapies has revolutionized the treatment of those cancers (8). The treatment landscape for patients with HER2-postive breast cancer is still evolving, with current options now including HER2-targeted mAbs, antibody–drug conjugates (ADC), and small-molecule tyrosine kinase inhibitors (TKI); however, patients’ disease often develops resistance and progresses on therapy. In addition, HER2-positive cancers have a propensity of developing brain metastases, and the historical paucity of effective treatments in this space has negatively impacted the longevity and quality of life of patients who manifest them (9). These unmet medical needs underscore the need for new treatment paradigms that can combat resistance, are tolerable, and are effective against brain metastases.

HER2, as a member of the ErbB family of receptor tyrosine kinases, comprises several domains, including its extracellular domain and intracellular kinase domain, which could both be targets for therapeutic intervention (4). Although HER2 has no known ligand, the homodimerization and heterodimerization of HER2 with other ErbB family receptors potentiates its kinase activity and results in the initiation of multiple signaling cascades that promote cell proliferation and inhibit apoptosis, including the MAPK and the PI3K-AKT pathways (3, 4, 6). HER2 overexpression results in a higher propensity of dimerization and thus results in constitutive activation of signaling pathways and dysregulated cell proliferation (10).

Recent combinatorial approaches to HER2 inhibition by two different HER2-targeted agents with complementary mechanisms of action have shown promising increased antitumor activity (11–13). Dual HER2 inhibition has been shown to improve pathologic responses or clinical outcomes over single-agent HER2 inhibition in patients with HER2-positive breast cancer (14–18). In line with these efforts, the combination of the anti-HER2 mAb trastuzumab and tucatinib, a potent, reversible, oral TKI that is highly selective for the kinase domain of HER2, resulted in more effective inhibition of HER2-mediated signaling and proliferation in preclinical studies, compared with each monotherapy (12). In translation to clinical practice, the combination of tucatinib, trastuzumab, and capecitabine has been shown to significantly improve outcomes over trastuzumab and capecitabine in patients with metastatic breast cancer, including those with active brain metastases (17, 19–21).

Given these clinical findings, additional therapeutic combinations inclusive of tucatinib are being evaluated clinically (22). One promising combination is tucatinib with T-DM1, an ADC comprising trastuzumab conjugated via a stable linker to DM1, a cytotoxic microtubule inhibitor. As an ADC, the activity of T-DM1 is dependent on its internalization and trafficking to the lysosome upon binding to the extracellular domain of HER2. Within lysosomes, proteolytic degradation of the ADC releases its cytotoxic payload, resulting in cell-cycle arrest and apoptotic cell death (23).

Herein, we report the antitumor activity of tucatinib combined with T-DM1 in preclinical breast cancer models, including T-DM1–resistant patient-derived xenografts (PDX). We also show the antitumor activity of tucatinib in an intracranial tumor model, addressing the potential of this therapeutic in often intractable metastatic lesions. By interrogating the combination in HER2-expressing cancer cell lines, we elucidate a novel mechanism by which tucatinib improves the cytotoxicity of T-DM1. We demonstrate that by increasing the levels of inactive HER2 at the cell surface, tucatinib drives increased ADC delivery and greater antitumor cytotoxicity.

## Materials and Methods

### Cell Culture and Cell Lines

All cell lines were obtained from ATCC or the Leibniz Institute (DSMZ). Cell lines were demonstrated to be free of *Mycoplasma* by PCR evaluation and were authenticated by marker analysis prior to banking (completed by IDEXX BioAnalytics). Cells were cultured at 37°C and 5% CO_2_ conditions, in appropriate growth medium as indicated, for a maximum of 7 weeks (7–15 passages; ref. 12). *Mycoplasma* testing was performed every 3 weeks while cells were in culture using Lonza MycoAlert *Mycoplasma* Detection Kit (#LT07-318).

BT-474::HA-Ubiquitin (Ub) and SK-BR-3::HA-Ub cell lines were established by transducing parental cells with a lentivirus that had an expression cassette of hemagglutinin (HA)-tagged ubiquitin from a human phosphoglycerate kinase promoter and a puromycin resistance gene. Cultures were enriched for stably transduced cells by the addition of 3 µg/mL puromycin in media for 3 weeks. Pooled transfected cells were evaluated and selected for equivalent HA-Ub expression.

### Cytotoxicity Assays and Drug Combination Analysis

Cytotoxicity assays were performed using CellTiter-Glo (CTG) Luminescent Cell Viability assay (Promega Corporation) as per the manufacturer's protocol. Cells were seeded in 384-well plates and incubated overnight as described previously (12). Cells were dosed in triplicate with combinations of 2-fold increasing concentrations of tucatinib (0.01–25,000 nmol/L) and 3-fold increasing concentrations of T-DM1 (0.01–25,000 ng/mL). Cell viability was measured at 96 hours after dosing.

Data for single-agent activity, or single-agent plus a fixed dose of a second agent, were analyzed in Prism (GraphPad Software) and IC_50_ (concentration required for 50% inhibition) and Emax (maximal effective concentration) values were generated from best-fit curves. Data reflecting the full matrix of dose combinations of both agents were analyzed to assess synergy or antagonism of each combination relative to the highest single agent (HSA) additivity model, using a previously described analytic and significance framework (24) and is described in further detail in the Supplementary Materials and Methods.

### Quantitative Flow Cytometry Analysis of Cell Surface HER2 Density

HER2 cell surface levels were determined by quantitative FACS (QFACS) analysis. Cells were harvested by Versene (Thermo Fisher Scientific) dissociation, washed twice in PBS, and stained with an anti-HER2 antibody (R&D Systems; #MAB1129) and Zombie Aqua (BioLegend; #423101) for dead cell exclusion. Cells were fixed with 1% paraformaldehyde in PBS prior to analysis. HER2 receptor copy number per cell was quantified using a QIFIKIT (Agilent Dako; #K0078) as per the manufacturer's protocol.

### HER2 Protein Expression Assays

Cells were plated in a 6-well format and treated with either 30 or 100 nmol/L tucatinib or lapatinib for either 24 or 48 hours and harvested into ice-cold lysis buffer [Cell Signaling Technology (CST); #9803] supplemented with 1:200 Protease Inhibitor Cocktail (Millipore Sigma; #535140) and 1:100 Phosphatase Inhibitor Cocktail (CST; #5870S). Protein concentration of lysates was measured using a bicinchoninic acid assay. Protein levels were analyzed using a Wes system (ProteinSimple) by loading samples for equal protein concentration. HER2 levels were determined using an anti-HER2 antibody (CST; #2165s) and normalized to GAPDH levels (CST; #2118). Cell surface expression of HER2 was measured by QFACS as described above.

### Ubiquitin Immunoprecipitation Assays

SK-BR-3 and BT-474 cells stably expressing HA-Ub were treated with either tucatinib, lapatinib, or neratinib (100 nmol/L) for 24 hours. MG132 proteosome inhibitor (10 µmol/L) was added 6 hours prior to cell harvest. Cell lysates were generated as described above. Ubiquitin was immunoprecipitated at 4°C overnight with an anti-HA antibody using the Pierce HA Tag IP/Co-IP Kit (Thermo Fisher Scientific) following the manufacturer's instructions, but with 25 µL of 0.1X ProteinSimple Sample Buffer (ProteinSimple) for elution. The eluate was probed for HER2 by Wes as described above.

### Protein Phosphorylation ELISAs

Phosphorylation of HER2 (pan-Tyr, #7968), HER3 (pan-Tyr, #7911), AKT (phos-AKT1, #7160C), and MEK (phos-MEK1/2, #7175C; all from CST) was determined using ELISAs. BT-474 cells (1.5 × 10^5^ cells per well in a 24-well plate) were seeded overnight and treated with tucatinib (30 nmol/L), T-DM1 (1 µg/mL), or tucatinib plus T-DM1 for 24 hours. Assays were run in triplicate with two internal replicates. Lysates were prepared with buffer containing protease and phosphatase inhibitors, and samples were processed using PathScan Sandwich ELISA kits (CST) as per the manufacturer's protocol. Phosphorylated protein levels were normalized to total protein levels (ErbB2, #7310; HER3, #7888C; AKT, #7170C; MEK, #7165C all from CST) and quantified relative to untreated cells. Data were plotted with SEM using Prism.

### HER2 Internalization Assays

For pulsed assays, SK-BR-3 cells were plated into 96-well flat-bottomed Ibidi plates (Ibidi GmbH) and adhered overnight at 37°C. Cells were incubated with either prechilled Alexa Fluor (AF) 488 fluorescently-labeled or quenched fluor-labeled trastuzumab (3 µg/mL resuspended in PBS) at 4°C for 20 minutes. Buffer was then removed, cells were washed once with PBS, and buffer was replaced with media with or without either tucatinib or neratinib (100 nmol/L). Cells were incubated at 37°C until the indicated timepoints. Cells were then fixed with Cytofix/Cytoperm buffer containing 4.2% formaldehyde (BD Pharmingen) and counterstained with Hoechst dye before being visualized using an IN Cell Analyzer high content fluorescence imaging system (Cytiva Life Sciences). Subsets of cells were also treated with chloroquine (10 µmol/L) to inhibit lysosomal degradation and counterstained for Lamp1 (BD Pharmingen; #562622). Images were processed using ImageJ software (imagej.net). Diagrams were generated with BioRender (https://biorender.com/).

For constant exposure Incucyte internalization assays, SK-BR-3 cells were plated into Corning 3603 96-well flat-bottomed plates (Corning) and adhered overnight at 37°C. Trastuzumab was conjugated to Incucyte fabfluor dye (Sartorius AG; #4722) according to the manufacturer's protocol. Media containing either Fabluor-labeled or quenched fluor-labeled trastuzumab (1 µg/mL), with or without either tucatinib or neratinib (30 nmol/L) was added to cells. Cells were transferred to an Incucyte Imager/Incubator system and fluorescence was monitored over time. Fluorescence intensity was analyzed using Incucyte software; the total fluorescence intensity of the well was divided by the area of confluence, as determined by simultaneous phase contrast imaging. Data were exported to and graphed using Prism.

### Mass Spectrometry Analysis of T-DM1 Catabolites

BT-474 cells (5 × 10^5^ cells/mL per condition) were plated and incubated overnight in media containing T-DM1 (1 µg/mL) and/or tucatinib or neratinib (30 nmol/L). For both intracellular and extracellular drug quantification, cells were centrifuged to isolate the cell pellet from supernatant. Untreated media and cell pellets were used as blank matrix to prepare standard curves. Spiking solutions in H_2_O/acetonitrile (ACN) were made using reference material of DM1 and Lys-MCC-DM1 (MedChemExpress) and spiked into untreated matrix to make an eight-point standard curve. Ansamitocin, as an internal standard, was added to all standards and samples. Samples were lysed in the presence of protease inhibitor cocktail using sonication, and underwent reduction, precipitation, and alkylation. Extracts were analyzed via LC/MS-MS using a Sciex 6500+ (AB Sciex) coupled to a Shimadzu LC-20AD (Shimadzu) with a Discovery C18 column (Supelco Discovery HS-C18, 2.1 × 50 mm, 3 µm; Sigma-Aldrich) with ACN plus 0.1% formic acid and water plus 0.1% formic acid used as mobile phases. For unknown concentrations, DM1 and Lys-MCC-DM1 analyte to internal standard peak area ratios were calculated and translated into concentrations using the standard curve. Results were averaged over three replicates and graphed using Prism. Additional method details are available in the Supplementary Materials and Methods.

### IHC

Tumor sample sections for IHC were stained on the IntelliPATH FLX automated stainer (Biocare Medical) for Ki67 (Abcam PLC; #ab15580), caspase-3 (CST; #9661T), HER2 (Biocare; #ACA342B), phospho-HER2 (CST; #2243S), phospho-HER3 (CST; #2842S), phospho-AKT (CST; #3787L), and phospho-ERK (CST; #4370L).

Slides were digitized at 20X magnification to produce whole slide images (WSIs). WSIs were computationally analyzed using HALO image analysis software v3.1.1076.283 (Indica Labs), which was trained to classify tumor versus stroma. Analysis of tumor markers, including phospho-epitopes, was confined to delineated tumor tissue. All classifications and image analysis markups were assessed visually to verify accuracy. Data were analyzed in Prism and one-way ANOVA performed followed by Dunnett multiple comparison test. Additional method details are available in the Supplementary Materials and Methods.

### Xenograft Models

Animal studies were conducted at Seagen or Champions Oncology (Rockville) Association for Assessment and Accreditation of Laboratory Animal Care International–accredited facilities, under Institutional Animal Care and Use Committee approval. Xenograft models (Seagen) were generated by subcutaneously implanting BT-474 cells or patient-derived (PDX) breast tumor fragments (CTG-0717, CTG-0708, and CTG-0807; Champions Oncology; obtained with written informed consent) into the flanks of athymic nude mice. Animals were treated with tucatinib (50 mg/kg orally, twice daily), T-DM1 or an IgG1-DM1 nonbinding control ADC (10 mg/kg intraperitoneally), tucatinib plus T-DM1, T-DM1 plus IgG1-DM1, or vehicle (30% Captisol, orally, daily). Animals were followed to the designated end of each experiment. BT-474 tumors were harvested from satellite animals after 7 days of dosing, 1–2 hours after final dose. Tumors were fixed in formalin for 18–24 hours and transferred to ethanol in preparation for IHC analysis. For tumor volume statistical analyses, *P* values were determined by a Mann–Whitney test comparing the tumor growth inhibition (TGI) values of tucatinib/T-DM1 combination to the closest single agent.

### Intracranial Tumor Model

The study was conducted at Seagen under Institutional Animal Care and Use Committee approval. BT474-Red Luciferase-expressing cells (250,000) were injected into the right striatum of nude mice bearing estrogen pellets (0.18 mg, 60-day release). Tumor size was monitored by either bioluminescence or MRI for 6–8 weeks until large enough for study enrollment. Tumor volume was measured via MRI at study initiation and twice weekly posttreatment. Mice were treated with vehicle, tucatinib (75 mg/kg orally twice a day for 56 doses), T-DM1 (10 mg/kg i.v. every 7 days for four doses) or in combination for 28 days. Survival was monitored for 56 days and plotted by Kaplan–Meier analysis. A cohort of satellite animals was sacrificed at 7 days, 2 hours after final dose. Whole brains were harvested, and tissue was fixed in formalin for IHC analysis as described above.

### Data Availability

The data generated in this study are available upon request from the corresponding author.

## Results

### Tucatinib Sensitizes HER2-positive Cancer Cells to T-DM1

**FIGURE 1:**
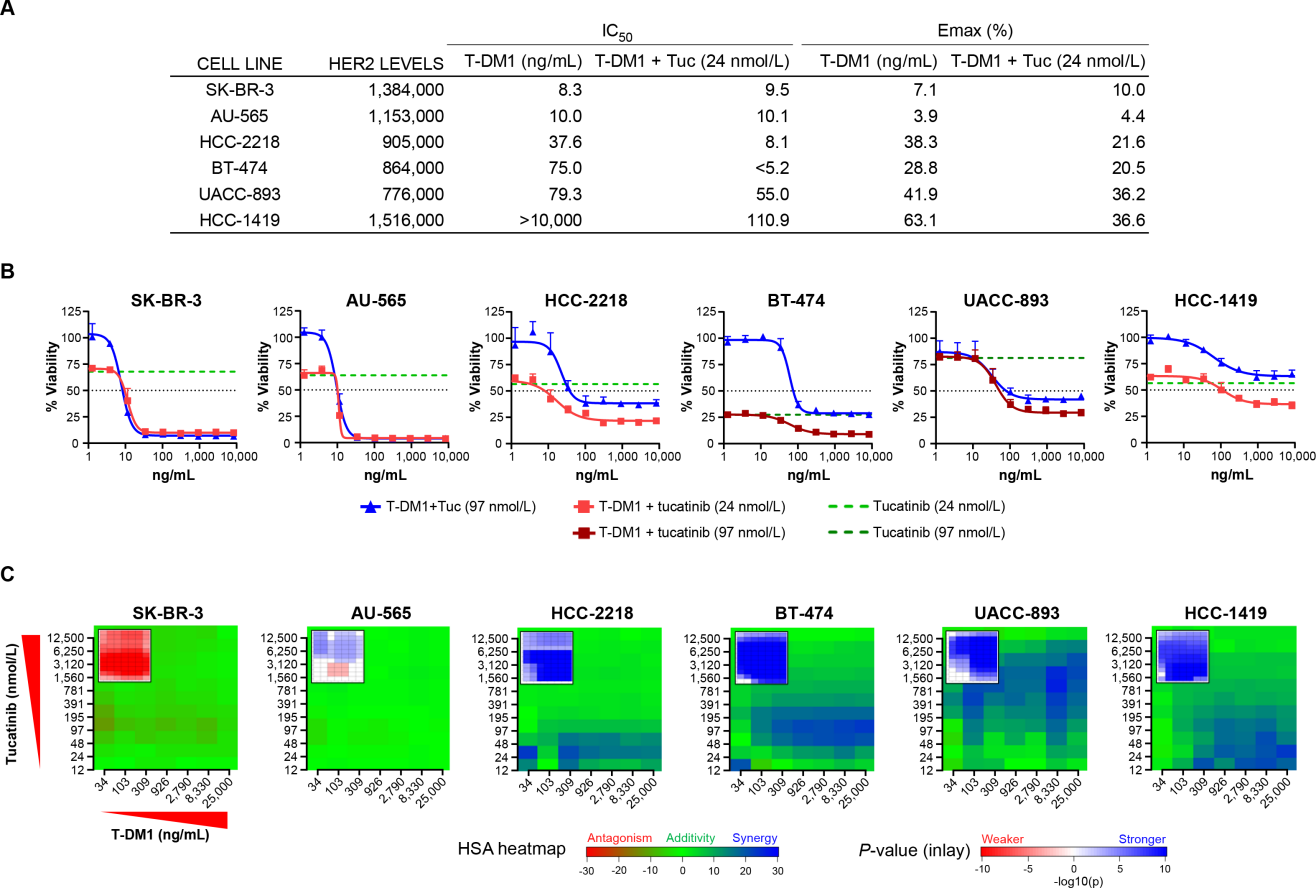
Tucatinib sensitizes HER2-positive cancer cells to T-DM1. **A,** Summary table of IC_50_ and Emax values for T-DM1 alone and in combination with tucatinib in CTG Luminescent Cell Viability cytotoxicity assays. **B,** CTG assays in which T-DM1 and tucatinib were screened in a panel of HER2-positive breast cancer cell lines. Data shown as mean + SD. **C,** Heat maps of matrixed CTG drug combination experiments testing the synergistic activity of tucatinib and T-DM1.

Dual inhibition of HER2 by tucatinib in combination with trastuzumab has been shown to yield more effective tumor suppression than each individual agent alone (12). To test the hypothesis that combining tucatinib with additional HER2-targeting agents with complementary mechanisms of action would further increase potency, we characterized the antitumor activity of tucatinib in combination with T-DM1. We first evaluated the cytotoxicity of each drug against a panel of HER2-positive breast cancer cell lines and observed a spectrum of sensitivity to T-DM1, with IC_50_ values ranging from highly sensitive (4.4–10 ng/mL; e.g., SK-BR-3 and AU-565) to insensitive (>10,000 ng/mL; e.g., HCC-1419; Fig. 1A and B). We then tested whether the addition of a clinically relevant dose of tucatinib (20–100 nmol/L; ref. 25) would increase the potency of T-DM1. In cell lines with diminished sensitivity to T-DM1 (HCC-1419, BT-474, UACC-893, and HCC-2218), cytotoxicity assays demonstrated an increase in antiproliferative activity for the combination of T-DM1 and tucatinib, with improvements in both IC_50_ and maximal efficacy (Emax) values (Fig. 1A and B). In contrast, more modest gains in improvement were observed in AU-565 and SK-BR-3 cells, which were already sensitive to T-DM1 monotherapy.

We further investigated the antiproliferative relationship between tucatinib and T-DM1 in combination. HSA model–based isobologram analysis across a matrix of titrated drug combination experiments illustrated the predominantly additive-to-synergistic nature of the improvement in cytotoxic activity of tucatinib and T-DM1 across a range of concentrations in cell lines with reduced sensitivity to T-DM1 (Fig. 1C), while additive improvements were noted in SK-BR-3 and AU-565 cell lines. The synergistic activity was dependent upon delivering the DM1 payload to cells via HER2 as determined by comparisons of tucatinib in combination with IgG1-DM1 nonbinding ADC, with which minimal synergy was observed (Supplementary Fig. S1).

### Tucatinib Mediates an Increase in HER2 through Reduced Ubiquitination of the Receptor

**FIGURE 2:**
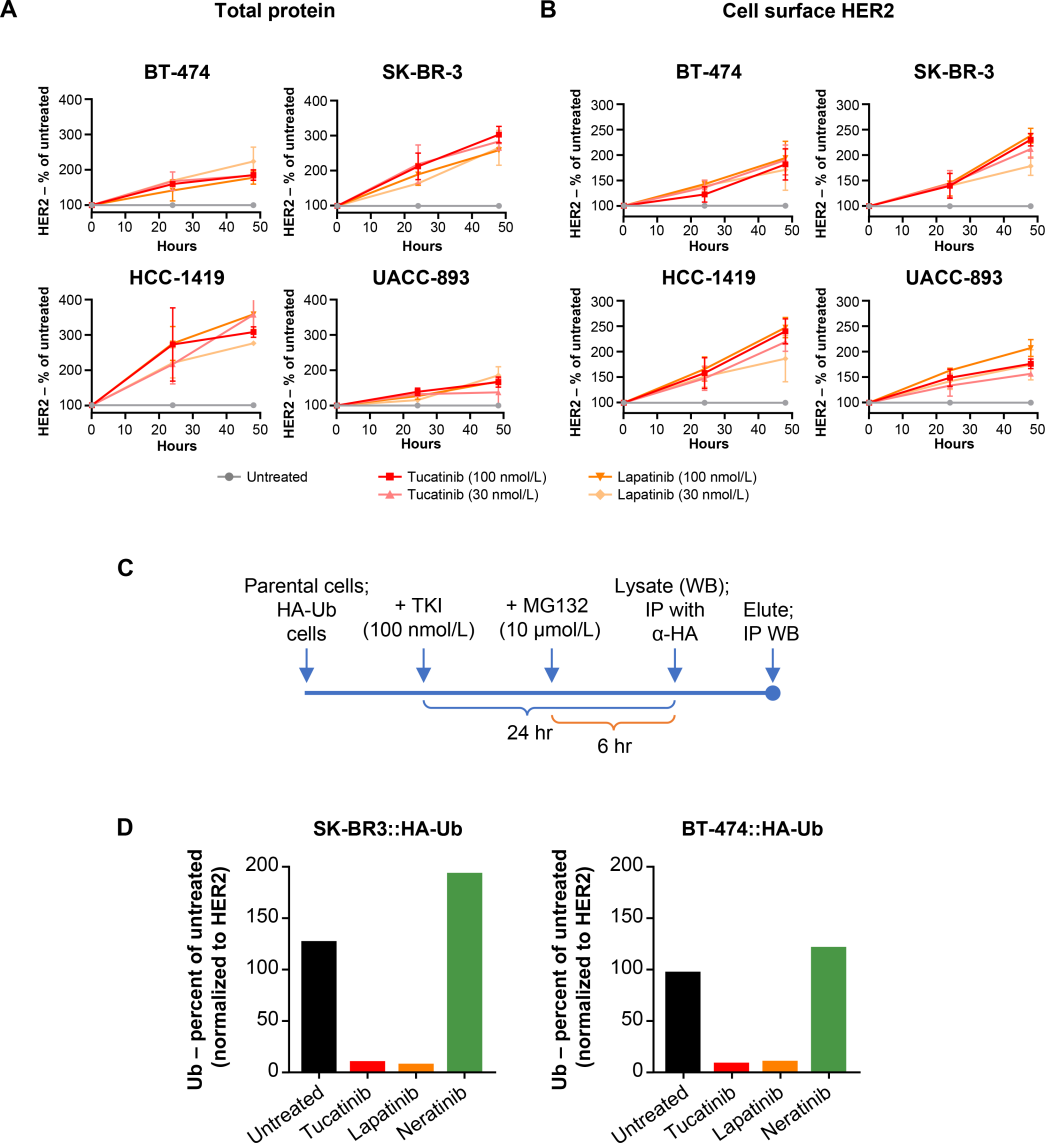
Tucatinib mediates an increase in HER2 through reduced ubiquitination of the receptor. QFACS of total (**A**) and cell surface (**B**) HER2 after treatment with tucatinib or lapatinib. Data shown as mean ± SEM. **C,** Schematic of IP assays/whole-exome sequencing analysis of stable HA-ubiquitin–expressing cell lines treated with TKIs. **D,** Analysis of HA-ubiquitin incorporated into HER2 (normalized to total HER2 protein levels). Results are representative of at least 2 independent experiments. IP, immunoprecipitation.

We sought to understand the underlying mechanism by which the combination of tucatinib and T-DM1 resulted in synergistic antiproliferative activity. As ErbB family-targeting TKIs have been reported to modulate HER2 protein levels and cell-surface localization (26–30), we hypothesized that tucatinib might alter HER2 protein levels. Accordingly, we investigated the impact of tucatinib on HER2 cell surface expression. Treatment of four HER2-positive breast cancer cell lines with tucatinib led to an increase in total and cell surface–localized HER2 protein levels over time (Fig. 2A and B; Supplementary Fig. S2A). This effect was similar to that observed with lapatinib, a reversible EGFR- and HER2-targeting TKI which has been shown to bolster antibody-dependent cellular cytotoxicity (ADCC) by stabilizing HER2 levels (29–31).

Because TKIs have been shown to modulate ubiquitin-mediated endocytic sorting and degradation (26–29), we then investigated whether tucatinib impacted HER2 ubiquitination. We engineered BT-474 and SK-BR-3 cell lines to express HA-tagged ubiquitin. Cells were treated with tucatinib, lapatinib, or neratinib (an irreversible TKI), followed by treatment with a proteosome inhibitor to block ubiquitin-mediated degradation prior to ubiquitin immunoprecipitation (Fig. 2C). Protein analysis demonstrated a reduction in ubiquitination of HER2 after treatment with tucatinib, with a similar effect seen following lapatinib treatment. In contrast, an increase in HER2 ubiquitination was observed following treatment with the nonreversible TKI neratinib (Fig. 2D; Supplementary Fig. S2B). These data support a mechanism by which tucatinib, a reversible TKI, promotes the stabilization of HER2 through the reduction of HER2 ubiquitination, in direct contrast with irreversible inhibitors such as neratinib, which promote the ubiquitination and degradation of HER2.

### Tucatinib Alters the Internalization Dynamics of HER2-targeted Antibodies

**FIGURE 3:**
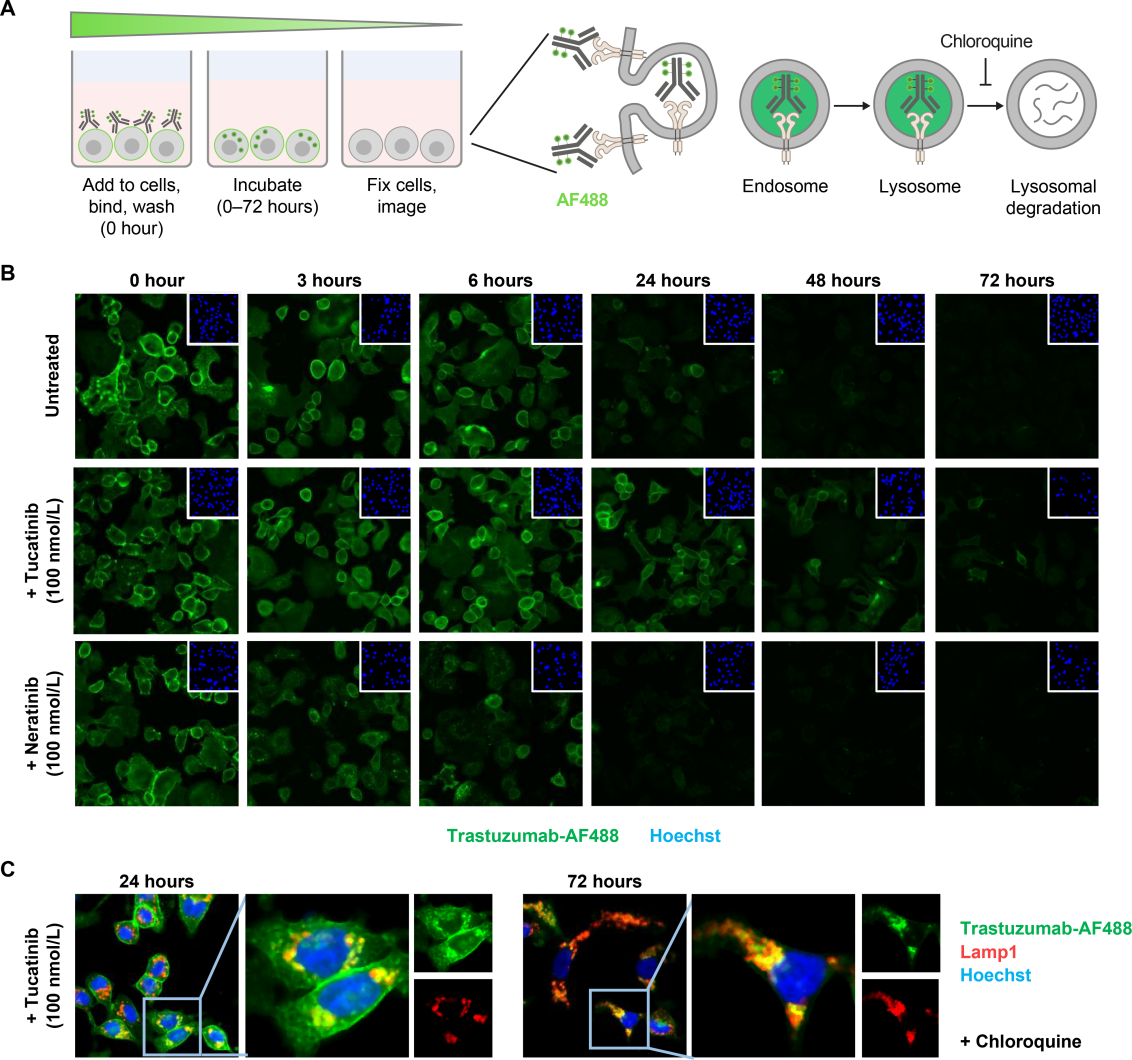
Tucatinib alters the internalization dynamics of HER2-targeted antibodies. **A,** Schematic of pulsed internalization assay with AF488 fluorescently-labeled trastuzumab. **B,** Images of SK-BR-3 cells in pulsed internalization assays incubated with AF488 fluorescently-labeled trastuzumab and/or treated with tucatinib or neratinib. Inlay images show counterstaining (Hoechst) to signify distribution of cells. **C,** Magnified images of tucatinib-treated cells in internalization assays, counterstained with lysosomal marker LAMP1. Chloroquine, which inhibits lysosomal function (49), was added to show more intense colocalization when lysosomal degradation is impaired. These results are representative of at least 2 independent experiments.

Our findings above and those of others suggest that reduction in ubiquitination is one mechanism by which HER2 appears to be stabilized at the cell surface by HER2-targeting TKIs (28–30). Consequently, it has been proposed that reduced HER2 ubiquitination can be prohibitive to its internalization (32). To assess the impact of tucatinib and reduced HER2 ubiquitination on the fate of cell-surface HER2, we evaluated HER2 trafficking dynamics by monitoring the localization and intensity of AF488-fluorescently-labeled trastuzumab in pulsed internalization assays (Fig. 3A). This allowed a labeled pool of HER2 at the cell surface to be observed over time. In untreated cells, trastuzumab-AF488 at the cell surface was reduced over the course of 6 hours, with little remaining by 24 hours. Treatment with neratinib led to a reduction in cell surface trastuzumab-AF488 levels as early as 3 hours, implying more rapid removal and depletion of HER2 from the cell surface, consistent with reported findings (Fig. 3B; ref 31). However, in tucatinib-treated cells, trastuzumab-AF488 was still clearly visible at the cell surface at 6 and 24 hours but was undetectable by 72 hours (Fig. 3B), implying a delay in internalization and/or degradation compared with neratinib.

Costaining with LAMP1, a lysosomal marker, demonstrated that in the presence of tucatinib, trastuzumab-AF488 localized to the lysosomal compartment in 24–72 hours time frame (Fig. 3C; Supplementary Fig. S3), suggesting that despite the reduction in ubiquitination, HER2 trafficked to the lysosome upon internalization. Confirmatory results were obtained from pulsed internalization assays utilizing trastuzumab labeled with a quenched fluor imaging reagent which fluoresces only upon processing in the lysosome (33), further demonstrating that HER2 can be internalized upon treatment with tucatinib (Supplementary Fig. S4A and S4B). Overall, these data suggest that in the presence of tucatinib, HER2 levels are stabilized at the cell surface, yet continue to be internalized and targeted to the lysosome after a short period of delay.

### Tucatinib Mediates Increased Internalization and Catabolism of T-DM1

**FIGURE 4:**
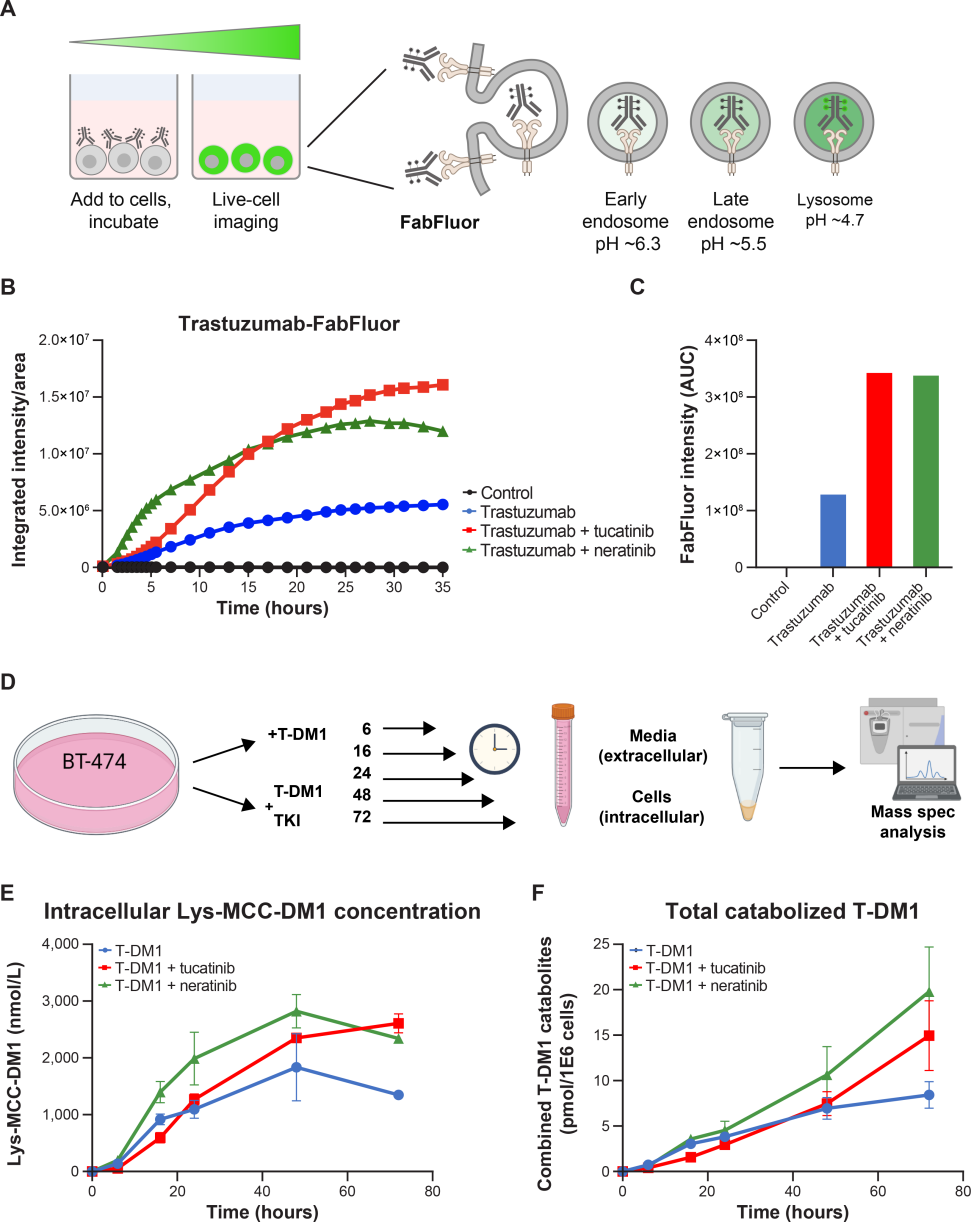
Tucatinib mediates increased internalization and catabolism of T-DM1. **A,** Schematic of constant exposure internalization assays with Fabfluor-labeled trastuzumab. **B,** Fluorescence intensity of SK-BR-3 cells in internalization assays imaged over time. **C,** AUC of fluorescence intensity in internalization assays calculated at 35 hours. Results in panels B and C are representative of at least 2 independent experiments. **D,** Schematic of T-DM1 catabolism analysis. **E,** LC/MS-MS analysis of the predominant intracellular catabolite of T-DM1, Lys-MCC-DM1, in BT-474 cells after treatment in the presence or absence of either tucatinib or neratinib. **F,** LC/MS-MS analysis of the sum of all T-DM1 catabolites, both in intracellular and extracellular fractions. Data in panels E and F shown as mean ± SEM. AUC, area under the curve.

One possible mechanism to account for the synergistic relationship between tucatinib and T-DM1 is through additional T-DM1 binding sites provided by elevated cell-surface HER2 levels, which could increase the overall delivery of T-DM1. To test this hypothesis, we employed constant exposure internalization assays in which cells were exposed to an unlimited pool of trastuzumab labeled with fabfluor, a dye that fluoresces upon reduction in pH (Fig. 4A). Fabfluor dye served as a localization indicator within the acidified compartments of endocytic vesicles and lysosomes, where T-DM1 is degraded, and the active cytotoxic molecule is released. Cells incubated with trastuzumab-fabfluor were imaged over time, and trastuzumab-fabfluor internalization was quantified in the presence or absence of TKIs. Constant exposure internalization assays demonstrated increased internalization of trastuzumab-fabfluor in the presence of either tucatinib or neratinib and reflected similar dynamics to trastuzumab-quenched fluor in pulsed assays, with rapid trastuzumab-fabfluor internalization observed with neratinib, and delayed internalization dynamics with tucatinib (Fig. 4B; Supplementary Fig. S4A and S4B). However, after a period of approximately 24 hours, neratinib-treated cells demonstrated an abatement of trastuzumab-fabfluor internalization, presumably because of concurrent degradation of HER2 receptors with increased ubiquitination that is mediated by neratinib. In contrast, trastuzumab-fabfluor internalization was sustained in the presence of tucatinib. Overall, continued measurement of internalization over a prolonged period (>30 hours) demonstrated that equivalent levels of trastuzumab-fabfluor ultimately accumulated in the lysosome in the presence of tucatinib or neratinib (Fig. 4C). Parallel experiments with quenched fluor-labeled trastuzumab demonstrated similar results (Supplementary Fig. S4C–S4E). These data suggest that while trastuzumab internalization initially occurs more rapidly upon treatment with neratinib, tucatinib treatment induces a more sustained delivery, likely due to more HER2 protein available for binding at the cell surface.

To correlate HER2 internalization to T-DM1 delivery and catabolism, we evaluated the effect of tucatinib on the intracellular accumulation of the cytotoxic payload of T-DM1. We treated BT-474 cells with T-DM1, with or without tucatinib or neratinib, for up to 72 hours, and then quantified T-DM1 catabolite concentrations using mass spectrometry (Fig. 4D). Of note, Lys-MCC-DM1 is the primary T-DM1 catabolite that is generated through lysosomal degradation and can saturate inside the cell (34, 35). Intracellular drug catabolism analysis reflected similar dynamics as observed with the internalization assays (Fig. 4E). Treatment with T-DM1 in combination with tucatinib led to increased intracellular concentration of Lys-MCC-DM1 over time, as well as an increase in all combined T-DM1 catabolites (DM1, MCC-DM1, Lys-MCC-DM1; intracellular and extracellular) compared with T-DM1 alone (Fig. 4E and F). Treatment with the combination of neratinib also led to an increase, with intracellular accumulation of Lys-MCC-DM1 occurring more rapidly initially but tapering off between 48 and 72 hours. In contrast, Lys-MCC-DM1 concentrations continued to increase in the presence of tucatinib during the same time frame (Fig. 4E). Collectively, these data support the conclusion that the increase in cell-surface HER2 associated with tucatinib mediates increased T-DM1 internalization and catabolism, leading to an elevated and sustained intracellular concentration of cytotoxic payload over T-DM1 alone.

### Increased Antitumor Activity is Observed *In Vivo* by Combining Tucatinib and T-DM1 in HER2-positive Breast Cancer Xenograft Models

**FIGURE 5:**
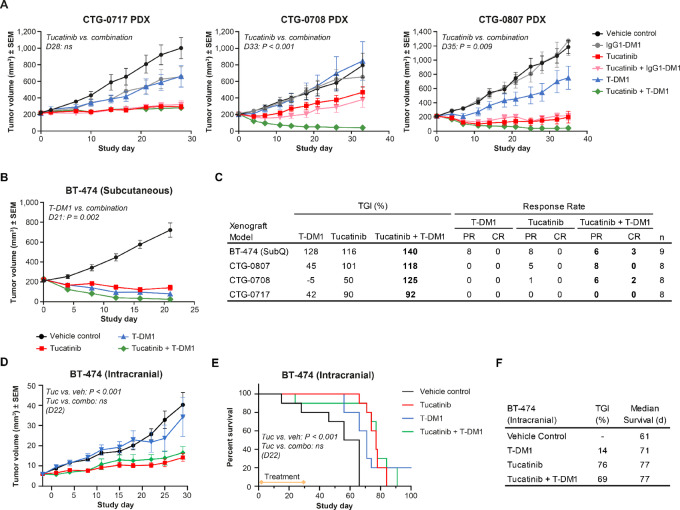
Combining tucatinib and T-DM1 in HER2-positive breast cancer xenograft models increases antitumor activity. Mean tumor volume over time in T-DM1–resistant breast cancer PDX models (**A**) and BT-474 CDX model (**B**). Tucatinib was administered orally at 50 mg/kg twice daily for the duration of the study, while T-DM1 and the IgG1-DM1 nonbinding control ADC were dosed at 10 mg/kg (single dose). **C,** Summary table of tumor growth inhibition and response rates of subcutaneous-implant xenograft models. **D,** Mean tumor volume over time in intracranially implanted BT-474-RedLuc xenograft model. **E,** Kaplan–Meier survival plots of intracranial BT-474-RedLuc xenograft model. **F,** Summary table of tumor growth inhibition and median survival days of intracranial BT-474-RedLuc xenograft model. All *P* values were determined by comparing the tucatinib/T-DM1 combination with the closest single agent. Data shown as mean ± SEM.

We wanted to determine whether the *in vitro* synergistic activity observed between tucatinib and T-DM1 would translate to improved antitumor outcomes. We therefore investigated the *in vivo* activity of tucatinib, T-DM1, and the combination of the two against three *HER2*-amplified breast cancer xenograft models derived from patients (PDX) that had shown clinical disease progression with trastuzumab combination regimens and were refractory to T-DM1 monotherapy *in vivo*. We also tested regimens in the BT-474 HER2-positive cell line–derived (CDX) xenograft model. Treatment of mice with tucatinib or the combination of tucatinib with T-DM1 was well tolerated with net positive weight gain over the course of the studies (Supplementary Fig. S5). In three out of the four models, treatment with tucatinib in combination with T-DM1 produced superior tumor growth suppression, demonstrating statistically significant combinatorial activity that was greater than either single agent (Fig. 5A–C). Importantly, the combination of tucatinib and T-DM1 resulted in more animals demonstrating partial or durable complete responses compared with treatment with either tucatinib or T-DM1 alone (Fig. 5C). In the outlier CTG-0717 PDX model, T-DM1 activity was comparable with the activity of IgG1-DM1 nontargeted control, suggesting that nonspecific uptake of the ADC was driving therapeutic response and that the model was not responsive to HER2-directed ADC drug delivery. The addition of tucatinib to T-DM1 in this model did not improve activity over either single agent. Given the inherent lack of HER2-directed ADC activity in this model, this finding is not incompatible with the hypothesis that increased combinatorial activity is driven by elevated HER2 levels mediated by tucatinib but does suggest that the combination may not overcome some types of inherent resistance to HER2-directed ADC activity. Collectively, the xenograft data are in alignment with *in vitro* observations that the combination of tucatinib and T-DM1 result in increased antitumor activity.

### Tucatinib can Suppress the Growth of Intracranial HER2-positive Tumors

The blood–brain/tumor barrier (BBTB) has been shown to impede delivery of effective concentrations of many anti-HER2 therapeutics into metastatic brain tumor tissue (36). However, tucatinib, in combination with trastuzumab and capecitabine, has shown promising clinical efficacy in patients with brain metastases, thus, we sought to evaluate the preclinical effect of tucatinib on the suppression of HER2-positive brain tumors. To address this, we developed an intracranial tumor model through the stereotactic implantation of BT-474 cells into the right striatum of the brain (Supplementary Fig. S6A). Radioactively-labeled tucatinib was detectable and enriched in the intracranial tumors (Supplementary Fig. S6B and S6C), suggesting tucatinib had effectively crossed the BBTB. Treatment of mice bearing intracranial tumors with a maximum tolerated dose (MTD) of tucatinib demonstrated that it was more effective at improving survival in comparison with vehicle or either lapatinib or neratinib (Supplementary Fig. S6D).

We evaluated the impact of tucatinib on the suppression of intracranial tumor growth in combination with T-DM1. A cohort of mice was implanted with intracranial tumors and administered either tucatinib, T-DM1, or the two in combination, for the length of 28 days. Suppression of intracranial tumor growth was observed in tucatinib-treated mice compared with mice treated with vehicle control. In contrast to its efficacy in the subcutaneous BT-474 model, treatment with T-DM1 did not appear to significantly impact tumors, suggesting limited exposure of T-DM1 in intracranial tissue. Consistent with this, intracranial tumor growth delay was similar in mice treated with tucatinib alone and in mice treated with tucatinib plus T-DM1 (Fig. 5D). After treatment was withdrawn, animals were further monitored for survival for a total of 100 days. Survival of mice with intracranial tumors was significantly longer after treatment with tucatinib or with the combination of tucatinib and T-DM1 compared with control or T-DM1 alone (Fig. 5E and F). Together, these data suggest that tucatinib is effective at suppressing the growth of intracranial tumors, but the full benefit of the combination with T-DM1 activity may be limited to tumors that are accessible to both agents.

### The Combined Suppression of HER2 Signaling by Tucatinib and T-DM1 is Associated with Decreased Tumor Growth

**FIGURE 6:**
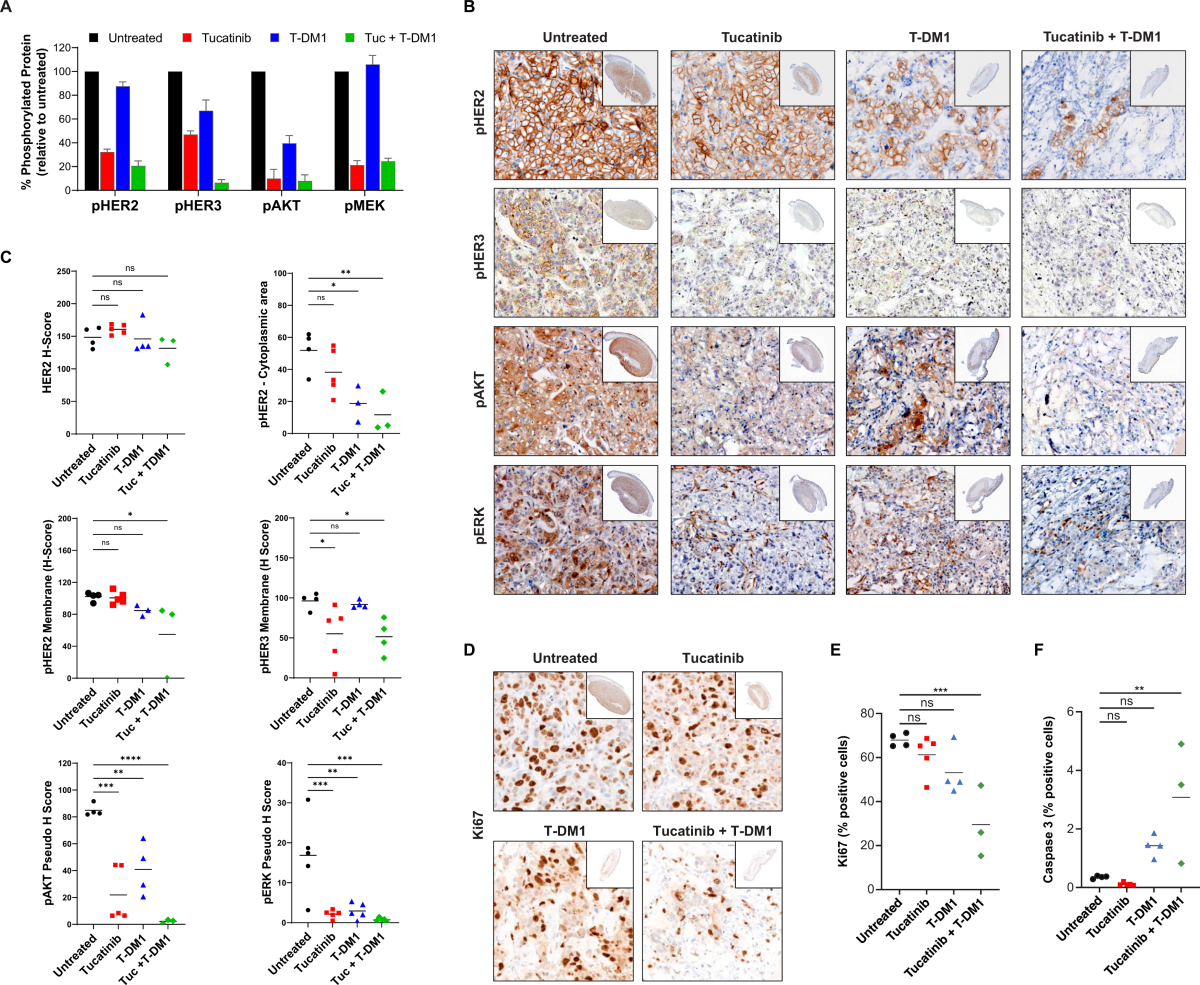
The combined suppression of HER2 signaling by tucatinib and T-DM1 is associated with reduced tumor growth. **A,** ELISAs quantifying phosphorylation of signaling components downstream of HER2 in BT-474 cells (as percentage phosphorylated protein vs. untreated cells with SEMs). **B,** IHC images of pHER2, pHER3, pAKT, and pMEK staining of the BT-474 xenograft tumor model. Inlay images represent HER2-stained tumor boundaries. **C,** HALO-based quantification of histologic analysis of HER2 and phospho-epitopes downstream of HER2 in the BT-474 tumor model**.** IHC images (**D**) and HALO-based quantification (**E**) of proliferation marker Ki67 in the BT-474 tumor model (7 days after dosing, 1–2 hours after final dose). Inlay images represent HER2-stained tumor boundaries. **F,** HALO-based quantification of histologic analysis of caspase-3 in the BT-474 tumor model. Results are representative of at least 2 independent experiments.

Elevated cell-surface HER2 levels may be beneficial for ADC drug delivery, but it is also possible that elevated cell-surface HER2 levels increase proliferative and antiapoptotic cues derived from HER2 signaling. Because both tucatinib and trastuzumab have been demonstrated to suppress HER2 signaling (12, 37), we wanted to assess the activation of HER2 after treatment with tucatinib, T-DM1, or the combination. We analyzed the phosphorylation status of the HER2 signaling pathway in BT-474 cells by ELISA (Fig. 6A). Despite overall higher levels of HER2, lower levels of phosphorylated HER2, HER3, AKT, and MEK were detected in cells treated with either tucatinib or T-DM1. Comparatively, tucatinib treatment suppressed the pathway to a greater extent than T-DM1, supporting its primary mechanism of action as a TKI. The combination of tucatinib and T-DM1 had an additive effect on the suppression of phosphorylated HER2 levels, suggesting that simultaneous inhibition of both the intracellular kinase and extracellular domains of HER2 with this dual HER2 inhibition strategy has the greatest impact on suppressing HER2 signaling, rendering additional HER2 molecules inactive.

Suppression of HER2 signaling was supported by *ex vivo* IHC analysis of tumor samples from a small subset of satellite animals (3–5 animals per group) of both the BT-474 subcutaneous model (Fig. 6B and C) and the intracranial model (Supplementary Fig. S6E). Phospho-epitope analysis demonstrated reduced HER2 pathway signaling in similar trends as the *in vitro* analysis, with greater reductions observed with the combination of tucatinib and T-DM1 than either agent alone, commensurate with suggested exposure (Fig. 6B and C). These data suggest the improved antitumor outcomes observed with the combination of tucatinib and T-DM1 are attributable to both an increase in cytotoxic payload delivery as well as further dampening of HER2 signaling.

Finally, we investigated the impact of increased suppression of HER2 signaling and increased delivery of cytotoxic payload on tumor cell survival and proliferation. IHC analysis of tissue samples harvested from the BT-474 subcutaneous and intracranial models demonstrated significantly reduced cell proliferation (Ki67) and increased apoptosis (caspase-3) in tumors after treatment with tucatinib in combination with T-DM1 compared with untreated controls (Fig. 6D–F; Supplementary Fig. S6F). These data confirmed that the combination of tucatinib and T-DM1 impedes the growth of HER2-positive tumors through the suppression of HER2-mediated proliferative signaling and promotes the death of HER2-positive tumor cells.

## Discussion

HER2-targeted therapies have dramatically improved treatment outcomes for patients with HER2-positive breast cancer (8). However, patients with HER2-positive metastatic breast cancer ultimately develop resistant tumors and experience disease progression (16, 38–40); thus, novel treatment strategies are needed to prolong survival. Combining agents with complementary mechanisms of action is an increasingly utilized approach for the treatment of cancer (16–18); for example, the use of two HER2-targeted agents with different mechanisms of action has demonstrated robust efficacy in clinical studies of patients with HER2-positive breast cancer (14–18). Combining tucatinib, a selective inhibitor for the kinase domain of HER2, with T-DM1, an ADC targeting HER2 for the delivery of a potent cytotoxic agent, is a new treatment regimen for consideration.

In our study, we tested the preclinical activity of tucatinib with T-DM1 and uncovered enhanced antitumor activity for the combination compared with the single agents. We demonstrated that tucatinib in combination with T-DM1 resulted in greater suppression of HER2 signaling and proliferation, and increased apoptosis of tumor cells. The combined effect between tucatinib and T-DM1 ultimately lead to synergistic cytotoxicity in HER2-positive breast cancer cell lines and increased antitumor activity in xenograft models, especially those with reduced sensitivity to T-DM1, compared with tucatinib or T-DM1 alone. Mechanistically, treatment with tucatinib reduced ubiquitination of the HER2 receptor, leading to an increase in cell surface HER2 levels. These results are consistent with previous studies demonstrating that lapatinib promotes HER2 receptor stabilization and accumulation at the plasma membrane, further enhancing immune cell–mediated ADCC when combined with trastuzumab (30), suggesting a common feature among reversible HER2-targeting TKIs (26). Extending these observations, we demonstrate that increased signaling-inhibited HER2 at the cell surface also mediates an increase in the internalization and catabolism of T-DM1. Consequently, a higher concentration of DM1 is delivered to cancer cells, resulting in improved directed tumor cell cytotoxicity without driving increased proliferation. These data support a novel regimen for the administration of an enhanced antitumor effect by targeting HER2 through two different modalities: tucatinib suppresses the intracellular kinase domain of HER2, while T-DM1 delivers a potent cytotoxic payload by binding to the extracellular domain of HER2. The combination of the two agents further suppresses HER2 signaling, and drives increased cytotoxic payload delivery.

The data we present focuses on the pairing of a reversible TKI with an ADC. Conversely, there have been studies demonstrating increased antitumor activity by pairing an ADC with neratinib, an irreversible pan-HER TKI, albeit through a contrasting mechanism: neratinib mediates increased ubiquitination of HER2, augmenting the targeting of ADCs to lysosome by increasing the rate of HER2 internalization (28, 32). The implication from these studies is that reduction of HER2 ubiquitination by reversible TKIs can impede the delivery of ADCs. We observed that initial HER2 internalization does occur more slowly during the first 24 hours upon treatment with tucatinib. However, we demonstrate that the kinetics of internalization is simply delayed, and that reduction of ubiquitination was ultimately not prohibitive to HER2 internalization. Moreover, treatment with tucatinib led to a greater level of inactive HER2 at the cell surface, potentiating increased ADC target engagement. Measurements of HER2 internalization over a period of several days rather than hours, in alignment with the half-life of T-DM1 in patients (41), showed that equivalent levels of HER2 were internalized after tucatinib treatment compared with neratinib. Consistent with increased T-DM1 internalization and lysosomal trafficking, we showed that combining tucatinib with T-DM1 increased intracellular levels of the cytotoxic DM1 payload, driving increased antitumor activity through reduced proliferation and increased apoptosis. In contrast, in the presence of neratinib, internalization of HER2 and catabolism of T-DM1 slowed down after 24 and 48 hours, respectively, most likely due to the removal of HER2 molecules from the surface followed by their consequent degradation. Collectively, these data support a novel mechanism for the administration of an enhanced antitumor effect by combining a reversible TKI with an ADC: tucatinib, by reducing HER2 ubiquitination levels, increased the cell surface availability of the ADC target, hence driving increased ADC delivery.

Brain metastases have, in the past, been managed with local therapy such as radiation and surgery, which is associated with neurocognitive adverse effects and poor quality of life (42, 43), highlighting an unmet medical need for novel targeted therapies with intracranial activity. In the HER2CLIMB clinical study, the treatment of patients harboring brain metastases with tucatinib in combination with capecitabine and trastuzumab showed longer overall survival, as well as higher intracranial objective response rate compared with patients treated with placebo in combination with capecitabine and trastuzumab (21). In our intracranial implant xenograft model, we show that tucatinib permeates the BBTB and accumulates in HER2-positive tumors. There, tucatinib inhibits HER2 pathway signaling, leading to a reduction in tumor cell proliferation and a delay in tumor growth, thus prolonging survival of mice bearing intracranial tumors. These observations of prolonged survival resemble those from HER2CLIMB and strongly support the intracranial activity and mechanism of tucatinib.

Surprisingly, in our intracranial xenograft model, the addition of T-DM1 to tucatinib did not appear to further inhibit intracranial tumor growth. It is important to note that, clinically, T-DM1 has demonstrated activity against brain metastases (44, 45). This may be due to changes in the tumor microenvironment and the loss of BBTB integrity, allowing T-DM1 to penetrate intracranial tumor tissue, as previously reported in other preclinical models (46, 47). However, while our *in vivo* model may not be entirely representative of the full clinical picture, it is supportive of the antitumor properties of tucatinib. Mechanistic modeling of the pharmacokinetics and target engagement of tucatinib in patients with cancer also provides further evidence that tucatinib could induce sufficient HER2 inhibition not only in brain metastases with a disrupted BBTB, but potentially also in micrometastases where the BBTB is largely intact (48).

On the basis of the results from our *in vivo* studies showing combinatorial activity with the addition of T-DM1 and potent intracranial activity in comparison with other TKIs, tucatinib may be the optimal TKI partner for other HER2-targeted therapies, particularly mAbs and ADCs. These translational results are especially noteworthy in the context of the clinical observations that the highly HER2-selective tucatinib is well tolerated, including in combination with T-DM1 (21). Our results support additional clinical studies of tucatinib in combination with T-DM1 in patients with HER2-positive metastatic breast cancer, in particular the ongoing HER2CLIMB-02 trial (NCT03975647).

## Supplementary Material

Supplementary Materials and Methods Supplementary Methods

Figure S1 Isobologram analysis of tucatinib with non-targeting IgG1-DM1 shows reduced synergy compared to T-DM1.

Figure S2 Tucatinib mediates an increase in HER2 through reduced ubiquitination of the receptor

Figure S3 HER2 in tucatinib-treated cells is targeted to the lysosome after a period of delay

Figure S4 Pulsed internalization assay with trastuzumab labeled with quenched fluor demonstrate increased internalization and lysosomal targeting with tucatinib

Figure S5 Effect of tucatinib, T-DM1, or combination treatment on mouse body weight in HER2+ xenograft models

Figure S6 Tucatinib suppresses growth of CNS tumors
