# Supplementary material for: HER2-Selective and Reversible Tyrosine Kinase Inhibitor Tucatinib Potentiates the Activity of T-DM1 in Preclinical Models of HER2-positive Breast Cancer
Source: Cancer Res Commun. 2023 Sep 25;3(9):1927–39. doi: 10.1158/2767-9764.CRC-23-0302 (PMC10519189; doi:10.1158/2767-9764.CRC-23-0302)
Supplement: Supplementary Materials and Methods — Supplementary Methods [file crc-23-0302-s01.docx]

# Supplementary Methods

## Drug combination analysis

Additivity models, such as HSA (highest single agent), give a prediction of additive activity (that is, neither synergy nor antagonism) for each dose combination in a matrix of all combinations, based on the single-agent activity of both drugs. Heatmap visualizations are used to depict the deviations of the observed viability for a given dose combination from the additive model predictions. Statistical significance of deviations from additivity is assessed using t-tests of model predictions minus observed viability. To increase power and reward consistency, tests are performed in 3×3 blocks of contiguous dose combinations. All 3×3 dose blocks tiling the dose combination space are tested, and separate heatmap visualizations are used to indicate 3×3 blocks that test as significant at a P-value of 0.01, adjusted for multiple testing by the total number of 3×3 blocks, in either direction (synergy or antagonism). A summary metric, mean significant deviation from additivity (MSDA) summarizes the overall synergy or antagonism in an experiment. MSDA is defined as the sum of (model prediction –observed viability) across all doses falling in any block tested as significant. The sum is normalized by the total number of dose combinations tested. MSDA > 0 indicates overall synergy; MSDA < 0, overall antagonism. Confidence intervals for MSDA values being significantly non-zero (positive or negative) have been computed based on *in silico* sham experiments combining one drug with itself, using historical data (1).

## Mass spectrometry analysis of T-DM1 catabolites

BT474 cells (5 × 10^5^ cells/mL per assay) were plated and incubated overnight in media containing T-DM1 (1 µg/mL) and/or 30 nM tucatinib or neratinib. For both intracellular and extracellular drug quantification, a known volume of cells was harvested by centrifugation to harvest both supernatant and cell pellet (750xg, 3 minutes, 4°C). After centrifugation, supernatant was collected and frozen at –80°C. Cells were washed 2x with an equal volume of ice-cold phosphate-buffered saline (PBS) and re-pelleted (750xg, 3 minutes, 4°C), and supernatant aspirated. This wash step was repeated, and the final pellet was stored in 100 µL of fresh PBS and frozen at –80°C. Untreated medium and cell pellets were used for standard curves and were prepared following the same sample processing procedures. Spiking solutions in H_2_O/ACN were made using reference material of DM1 and Lys-MCC-DM1 (MedChemExpress) and spiked into untreated matrix to make an eight-point standard curve (0.128–10,000 nM for cells and 1.64–1,000 nM for media). Ansamitocin as an internal standard was added to all standards and samples.

All samples and standards (cell pellet and media) were treated with PBS containing 1x protease inhibitor cocktail (Millipore Sigma; #535140). Samples were then vortexed, sonicated, and maintained on ice, lysing the cells. Following cell lysis, samples went through a reduction utilizing 20 µL of 5 mM tris (2-carboxyethyl) phosphine (Millipore Sigma; #646547-10×1mL) and incubated at 37°C for approximately 15 minutes. Protein precipitation was performed as follows: 450 µL of cold acetonitrile was added to samples, then vortexed for approximately 5 minutes at 4°C and centrifuged at 2000 × g for 5 minutes at 4°C. Following protein precipitation, samples were alkylated utilizing 30 µL of 100 nM N-ethylmaleimide (Millipore Sigma; #04259-5G) and incubated at 37°C for approximately 30 minutes. The corresponding supernatant was dried down under nitrogen.

Cell pellet and media extracts were reconstituted in 5% acetonitrile with 0.1% formic acid and were examined by LC-MS/MS using a Sciex 6500+ (AB Sciex LLC) coupled to a Shimadzu LC-20AD (Shimadzu). Extracts were injected onto a Discovery C18 column (Supelco Discovery HS-C18, 2.1 × 50 mm, 3 µm) with ACN plus 0.1% formic acid and water plus 0.1% formic acid used as mobile phases. DM1 and Lys-MCC-DM1 concentrations were determined using an MRM that selectively monitors for transitions specific to each small molecule (845.2 *m/z* to 485.3 *m/z* [precursor and fragment ion of DM1] and 1103.4 *m/z* to 485.3 *m/z* [precursor and fragment ion of Lys-MCC-DM1]) and the internal standard (635.5 *m/z* to 547.1 *m/z* [precursor and fragment ion of Ansamitocin]) DM1 and Lys-MCC-DM1 peak area ratios are calculated and translated into concentrations as a function of the standard curve using a linear regression. The results were averaged over three replicates and graphed using Prism.

## Immunohistochemistry

Tumor samples were cut into 4 µm sections onto adhesive slides and de-paraffinized in graded ethanol to distilled water. Slides were placed into the Decloaking Chamber™ NxGen (Biocare Medical) for retrieval in either Borg or Diva using the 110 ºC default program. Slides were stained on the intelliPATH FLX^®^ automated stainer (Biocare Medical). Tissues were stained for Ki67 (Abcam PLC), caspase-3, phospho-HER2, and phospho-ERK (all from CST). Sections were quenched with 3% H_2_O_2_ for 5 minutes, incubated in protein block for 10 minutes, with primary antibody for 1 hour, with secondary anti-rabbit horseradish peroxidase polymer for 30 minutes, and with chromogen for 5 minutes, with tris-buffered saline, 0.1% tween washes in between each incubation. Hematoxylin stain was applied for 5 minutes.

For visualization, slides were digitized at 20X magnification to produce a whole slide scan (WSI) on a Polaris scanner (Akoya BioSciences^®^). WSIs were computationally analyzed using HALO^®^ image analysis software v3.1.1076.283 (Indica Labs) to quantify the immunohistochemistry staining. A random forest classifier was trained to determine tumor versus stroma, and marker expression was determined in each location. Algorithms for membrane staining and cytoplasmic staining were developed to determine H-scores and pseudo H-scores (and positive-percentage cells) respectively. All classifications and image analysis markups were assessed visually to verify accuracy. Data were analyzed in Prism. A one-way analysis of variance was used followed by Dunnett’s multiple comparison test.

## Intracranial tumor model

One million BT-474 cells were injected into the brain of nude mice bearing estrogen pellets (0.5 mg, 35-day release). The mice were treated with vehicle (BID), Tucatinib (75 mg/kg, BID), Lapatinib (50 mg/kg, QD) or Neratinib (40 mg/kg, BID) starting 2 days after intracranial implantation of cells. Dose concentrations were selected based on maximum tolerated dosage. Treatment continued for the duration of the study (58 days) and mice were observed for neurologic symptoms or body weight loss. Survival was determined by Kaplan-Meier analysis. At the conclusion of the study, 69.2% of mice treated with tucatinib survived. Mice treated with lapatinib and neratinib showed less survival benefit (7.7% and 23.1%, respectively).

For ^14^C radioactivity measurements, gadopentetate dimeglumine contrast agent was injected intravenously at 0.4 mmol/kg via the caudal vein. Mice were treated with 70 mg/kg ^14^C-labeled tucatinib by oral gavage 12 hours apart for a total of three doses. Three hours post final dose, animals were sacrificed and whole brain tissue were harvested. Serial sections (4 mm) were bisected along the left and right hemispheres. ^14^C radioactivity (DPM/g tissue) was quantified from tumor- and non-tumor-bearing brain sections and was compared to radioactivity counted in plasma (DPM/mL) to determine the ratio of tissue to plasma distribution (2).

References

1. Thurman B, Rohm R, Arthur B. Novel framework for quantifying synergy in high-throughput drug combination cytotoxicity experiments. *Clin Cancer Res* 2020;**80**:835.

2. Zimmer AS, Van SAED, Anders CK. HER2-positive breast cancer brain metastasis: A new and exciting landscape. *Cancer Rep (Hoboken)* 2022;**5**(4):e1274 doi 10.1002/cnr2.1274.
