# Supplementary material for: HER2-Selective and Reversible Tyrosine Kinase Inhibitor Tucatinib Potentiates the Activity of T-DM1 in Preclinical Models of HER2-positive Breast Cancer
Source: Cancer Res Commun. 2023 Sep 25;3(9):1927–39. doi: 10.1158/2767-9764.CRC-23-0302 (PMC10519189; doi:10.1158/2767-9764.CRC-23-0302)
Supplement: Figure S1 — Isobologram analysis of tucatinib with non-targeting IgG1-DM1 shows reduced synergy compared to T-DM1. [file crc-23-0302-s02.docx]

## y
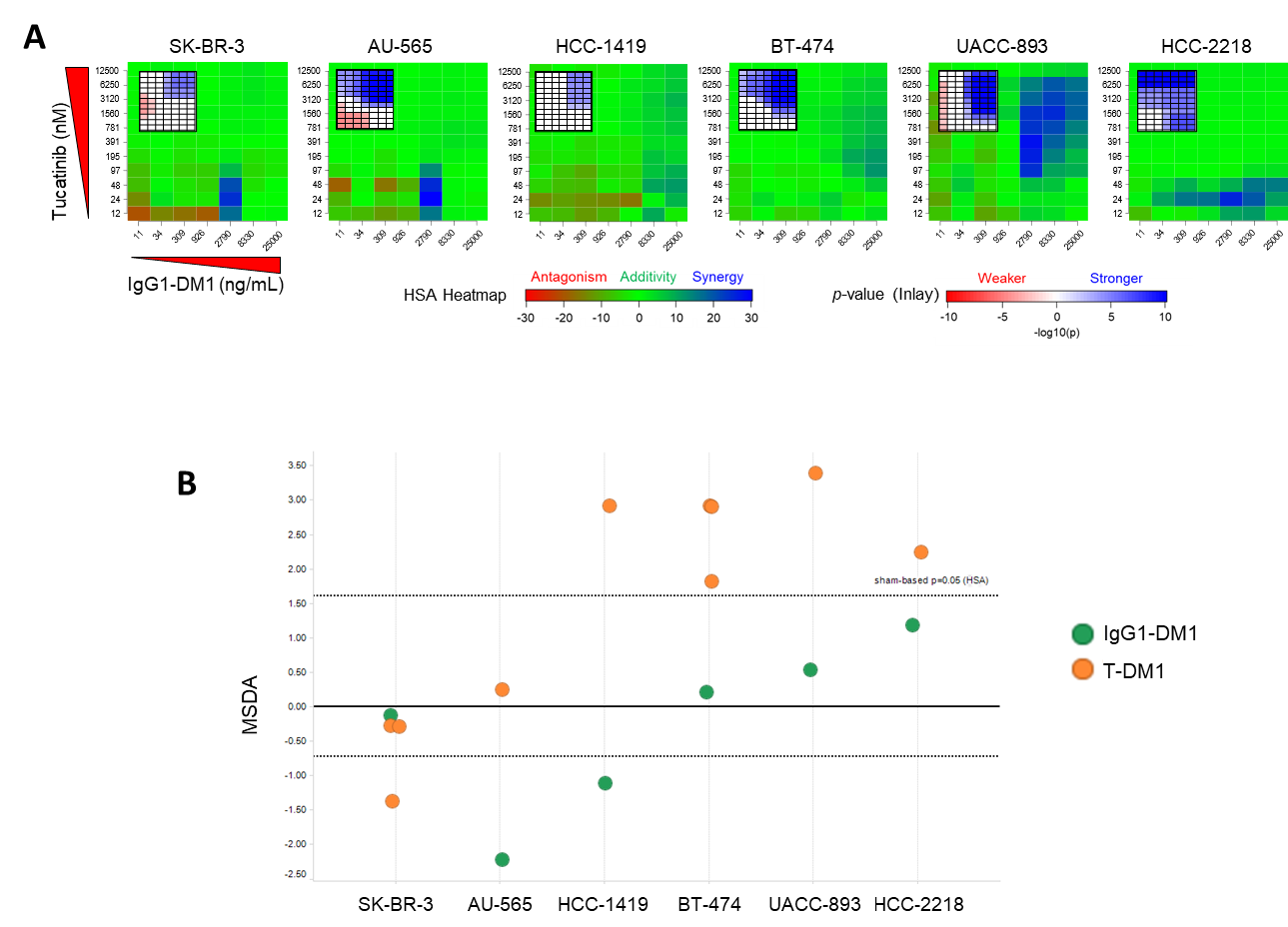
Supplementary Figure 1. Isobologram analysis of tucatinib with non-targeting IgG1-DM1 shows reduced synergy compared to T-DM1.

Drug combination analysis of 96-hour CellTiter-Glo Luminescent Cell Viability cytotoxicity assays. **A,** Combinatorial activity was evaluated for tucatinib and the antibody-drug conjugate IgG1-DM1 and for tucatinib. A drug concentration range spanning 12 nM–25 µM for tucatinib and 0.01 ng/mL–25 µg/mL for IgG1-DM1 was tested. Heatmaps indicate model predictions minus observed viability for each dose combination based on the HSA (highest single agent) additivity model, with color shading from blue to green to red indicating synergy, additivity, and antagonism, respectively, relative to the HSA model. Inset panels reflect significance of differences relative to the HSA model, measured in 3x3 blocks tiling the dose combination space. Blocks tested as significant at adjusted P < 0.01 are highlighted, with shades of blue indicating significance in the direction of synergy, and shades of red indicating significance in the direction of antagonism. **B,** Mean significant deviation from additivity (MSDA) values for each of the six combinations in panel (A), as well as the combinations with T-DM1 from **Figure 1** in the same cell lines. An MSDA value summarizes the overall synergy (positive MSDA) or antagonism (negative MSDA) in a combination experiment. The dashed lines indicate the 95% confidence interval for MSDA values being significantly non-zero (positive or negative) based on *in silico* sham experiments combining one drug with itself using historical data, as described previously (1). The graph shows that synergy with T-DM1 and greater than that with IgG1-DM1

## Reference

1. Thurman B, Rohm R, Arthur B. Novel framework for quantifying synergy in high-throughput drug combination cytotoxicity experiments. *Clin Cancer Res* 2020;**80**:835.
