# Supplementary material for: HER2-Selective and Reversible Tyrosine Kinase Inhibitor Tucatinib Potentiates the Activity of T-DM1 in Preclinical Models of HER2-positive Breast Cancer
Source: Cancer Res Commun. 2023 Sep 25;3(9):1927–39. doi: 10.1158/2767-9764.CRC-23-0302 (PMC10519189; doi:10.1158/2767-9764.CRC-23-0302)
Supplement: Figure S2 — Tucatinib mediates an increase in HER2 through reduced ubiquitination of the receptor [file crc-23-0302-s03.docx]

##
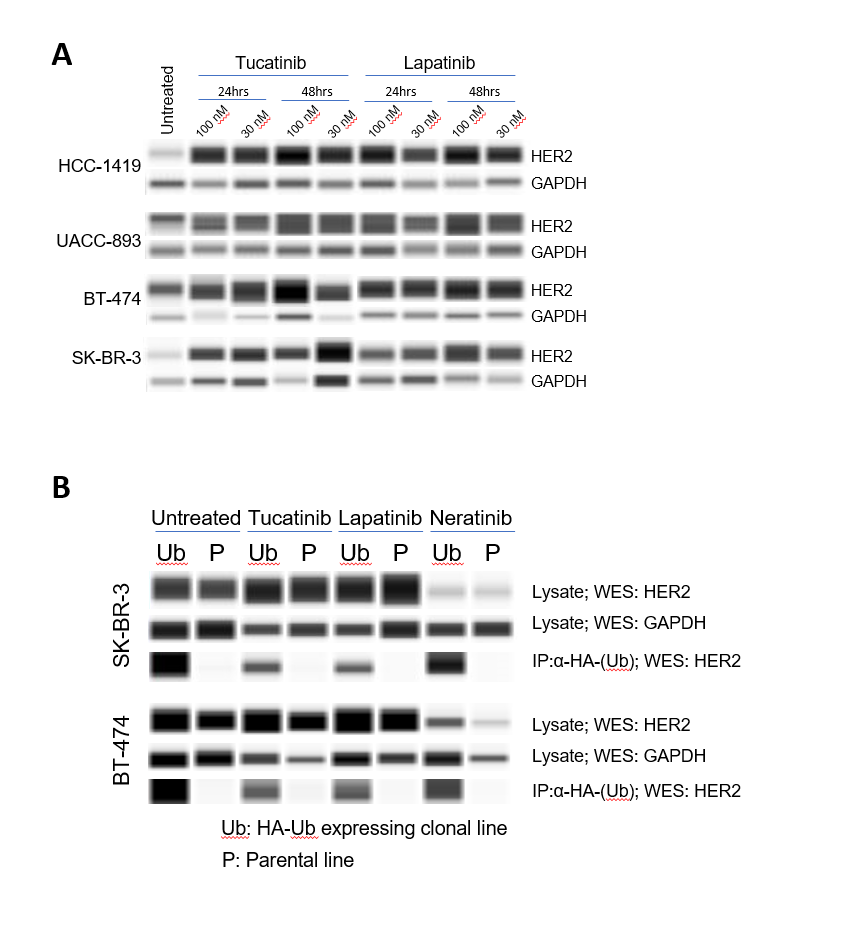


## Supplementary Figure 2. Tucatinib mediates an increase in HER2 through reduced ubiquitination of the receptor.

**A.** WES analysis of HER2 protein in lysates of breast cancer cell lines after treatment with tucatinib or lapatinib. **B.** WES analysis of IP assays from cell lines stably expressing HA-ubiquitin. Lysates were produced from cells treated with TKIs. Lysates were immunoprecipitated for HA and analyzed for HER2 pull-down.
