# Supplementary material for: HER2-Selective and Reversible Tyrosine Kinase Inhibitor Tucatinib Potentiates the Activity of T-DM1 in Preclinical Models of HER2-positive Breast Cancer
Source: Cancer Res Commun. 2023 Sep 25;3(9):1927–39. doi: 10.1158/2767-9764.CRC-23-0302 (PMC10519189; doi:10.1158/2767-9764.CRC-23-0302)
Supplement: Figure S3 — HER2 in tucatinib-treated cells is targeted to the lysosome after a period of delay [file crc-23-0302-s04.docx]

##
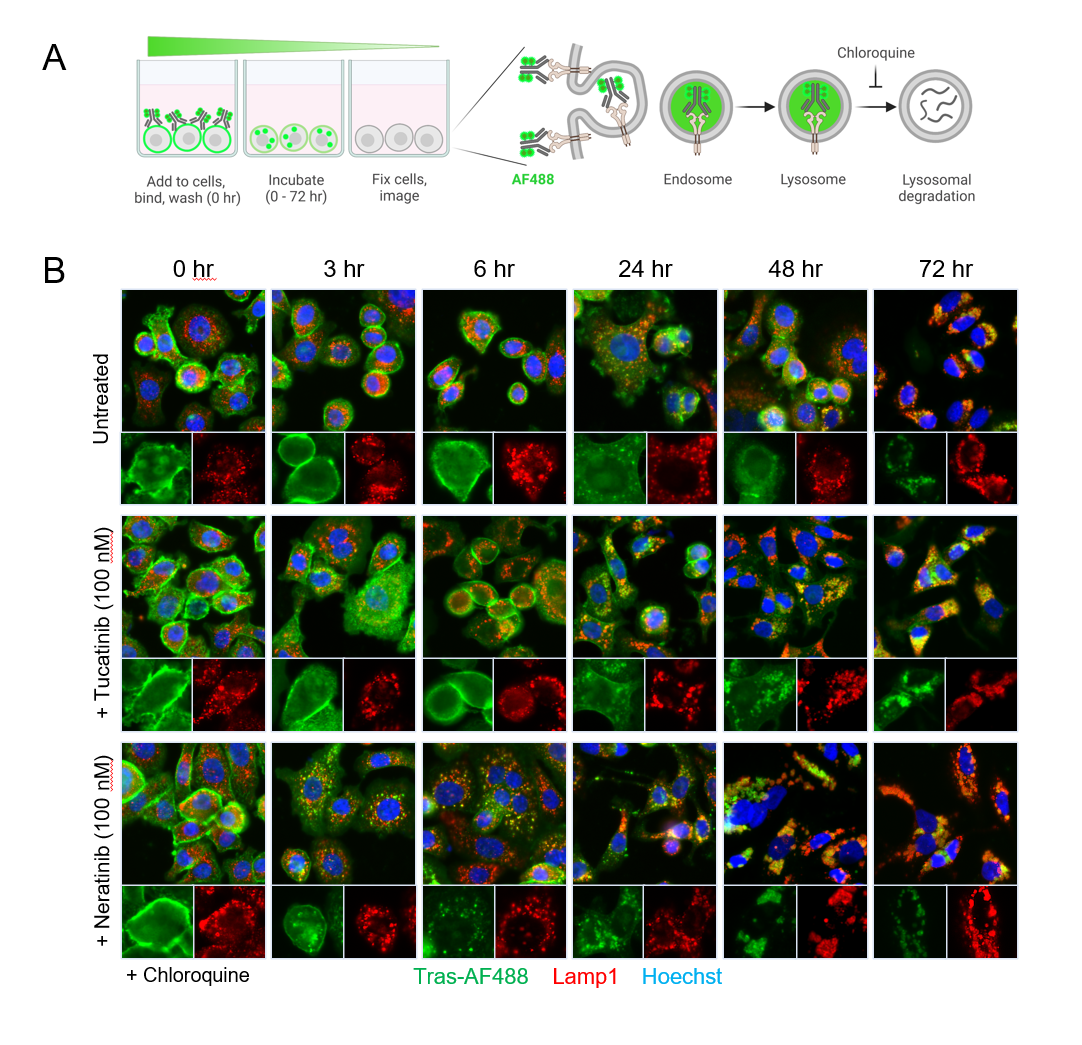
Supplementary Figure 3. HER2 in tucatinib-treated cells is targeted to the lysosome after a period of delay.

**A.** Schematic of pulsed internalization assay with AF488-fluorescently labeled trastuzumab. **B.** Images of SK-BR-3 cells in pulsed internalization assays incubated with AF488-fluorescently labeled trastuzumab and/or treated with tucatinib or neratinib. Hoechst staining of nuclei was used to illustrate the placement of individual cells while counterstaining with Lamp1 was utilized to mark the distribution of lysosomes. Magnified images (below) denote the overlap between trastuzumab-AF488 and the lysosomes at the given time points. Chloroquine, which inhibits lysosomal function (1), was added to show more intense colocalization when lysosomal degradation is impaired.

Reference

1. Mauthe M, Orhon I, Rocchi C, Zhou X, Luhr M, Hijlkema KJ*, et al.* Chloroquine inhibits autophagic flux by decreasing autophagosome-lysosome fusion. *Autophagy* 2018;**14**(8):1435-55 doi 10.1080/15548627.2018.1474314.
