# Supplementary material for: HER2-Selective and Reversible Tyrosine Kinase Inhibitor Tucatinib Potentiates the Activity of T-DM1 in Preclinical Models of HER2-positive Breast Cancer
Source: Cancer Res Commun. 2023 Sep 25;3(9):1927–39. doi: 10.1158/2767-9764.CRC-23-0302 (PMC10519189; doi:10.1158/2767-9764.CRC-23-0302)
Supplement: Figure S4 — Pulsed internalization assay with trastuzumab labeled with quenched fluor demonstrate increased internalization and lysosomal targeting with tucatinib [file crc-23-0302-s05.docx]

##
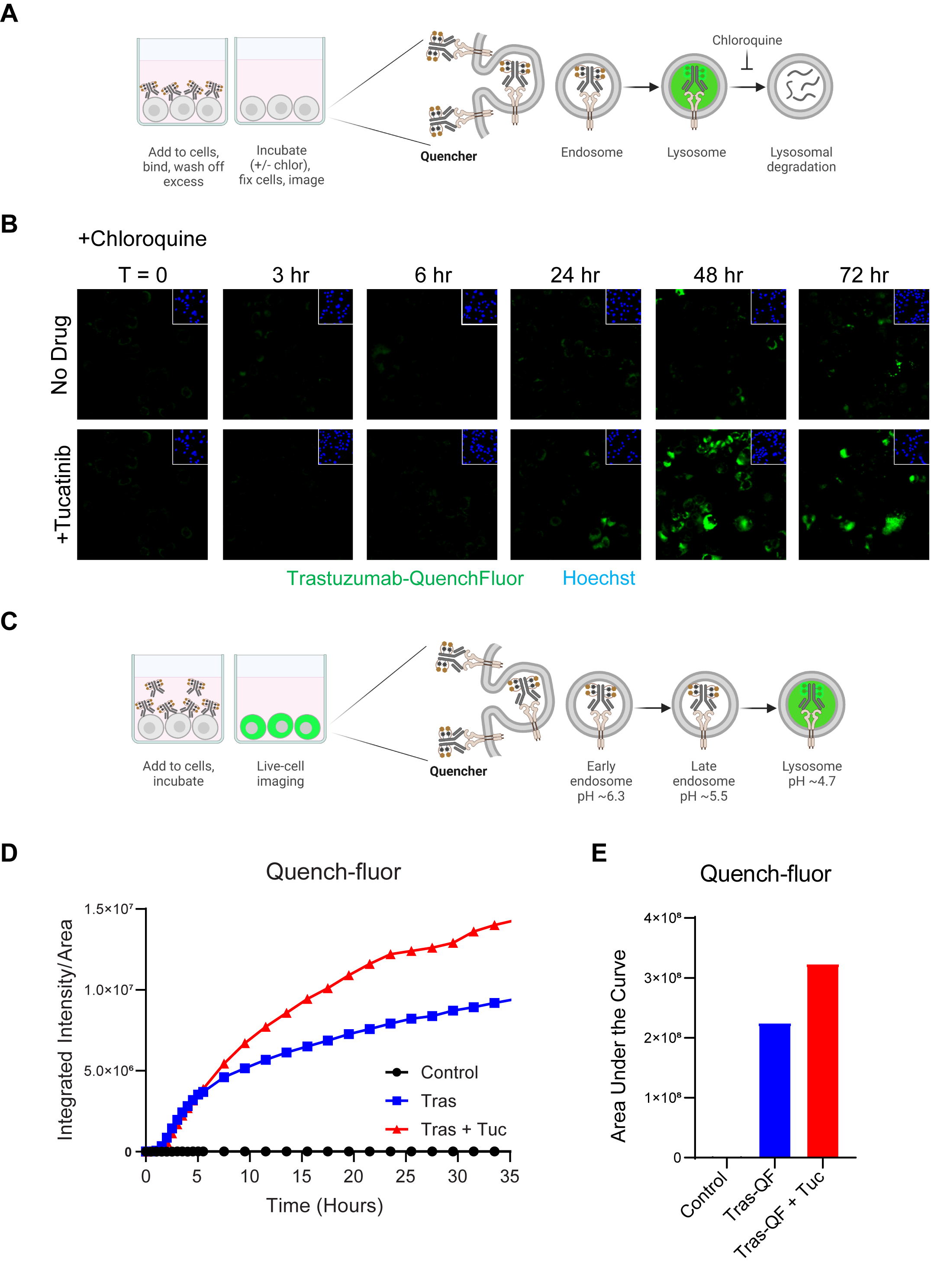
Supplementary Figure 4. Pulsed internalization assay with trastuzumab labeled with quenched fluor demonstrate increased internalization and lysosomal targeting with tucatinib.

**A,** Schematic of pulsed internalization assay with trastuzumab labeled with a quenched fluor, which fluoresces only upon processing in the lysosome. **B,** Images of SK-BR-3 cells in pulsed internalization assays incubated with trastuzumab labeled with a quenched fluor and/or treated with tucatinib or neratinib. Inlay images show counterstaining (Hoechst) to signify distribution of cells. **C,** Fluorescence intensity in constant exposure internalization assays imaged over time. **D,** Area under the curve of fluorescence intensity in internalization assays calculated at 35.5 hours.
