# Supplementary material for: HER2-Selective and Reversible Tyrosine Kinase Inhibitor Tucatinib Potentiates the Activity of T-DM1 in Preclinical Models of HER2-positive Breast Cancer
Source: Cancer Res Commun. 2023 Sep 25;3(9):1927–39. doi: 10.1158/2767-9764.CRC-23-0302 (PMC10519189; doi:10.1158/2767-9764.CRC-23-0302)
Supplement: Figure S5 — Effect of tucatinib, T-DM1, or combination treatment on mouse body weight in HER2+ xenograft models [file crc-23-0302-s06.docx]

##
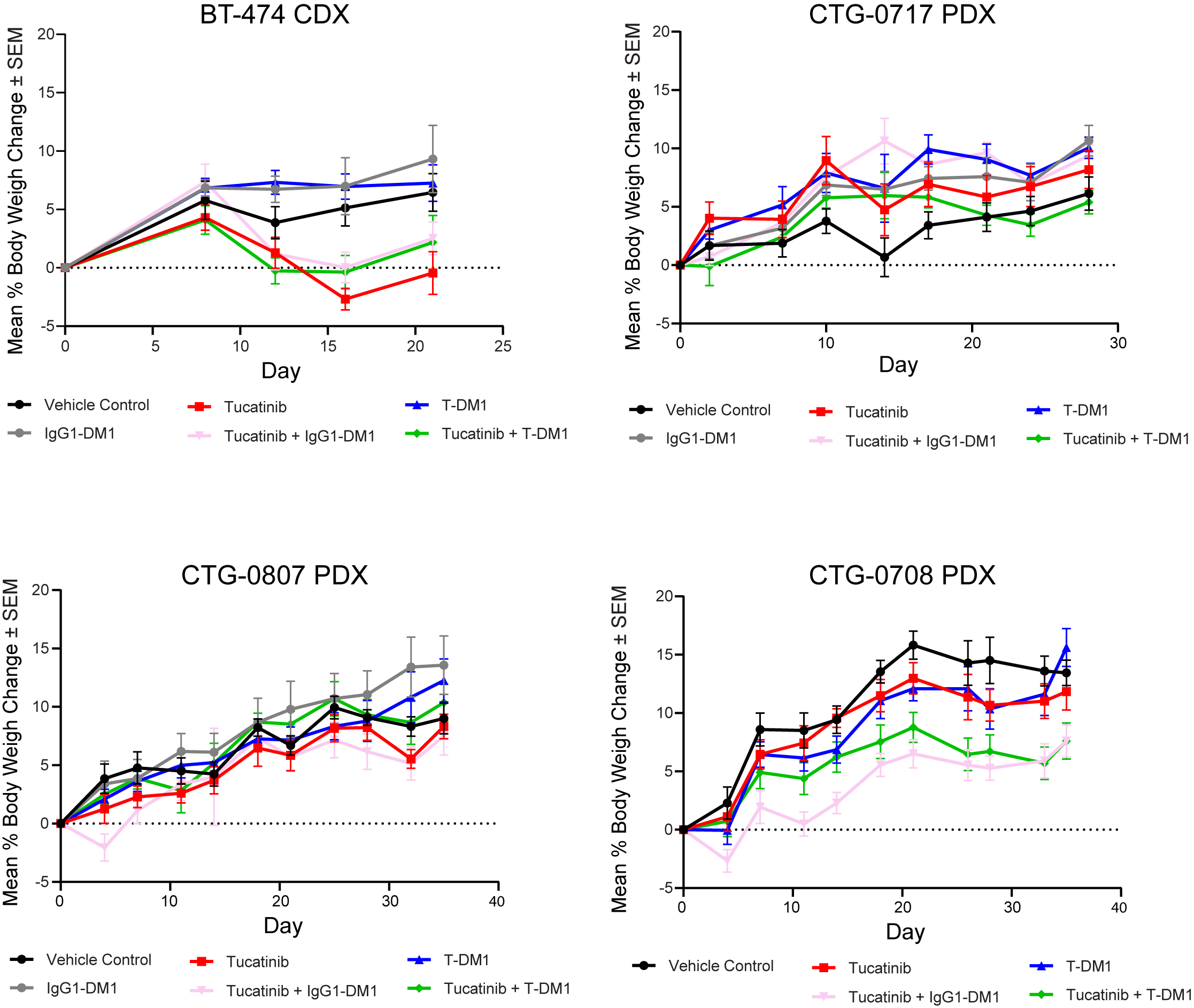


## Supplementary Figure S5. Effect of tucatinib, T-DM1, or combination treatment on mouse body weight in HER2+ xenograft models.

Percent body weight change in mice implanted subcutaneously with BT-474 cells and T-DM1-resistant xenografts. Tucatinib was administered orally at 50 mg/kg twice daily for the duration of the study, while T-DM1 and the IgG1-DM1 nonbinding control ADC were dosed at 10 mg/kg (single dose). Error bars represent ± SEM.
