# Supplementary material for: HER2-Selective and Reversible Tyrosine Kinase Inhibitor Tucatinib Potentiates the Activity of T-DM1 in Preclinical Models of HER2-positive Breast Cancer
Source: Cancer Res Commun. 2023 Sep 25;3(9):1927–39. doi: 10.1158/2767-9764.CRC-23-0302 (PMC10519189; doi:10.1158/2767-9764.CRC-23-0302)
Supplement: Figure S6 — Tucatinib suppresses growth of CNS tumors [file crc-23-0302-s07.docx]

##
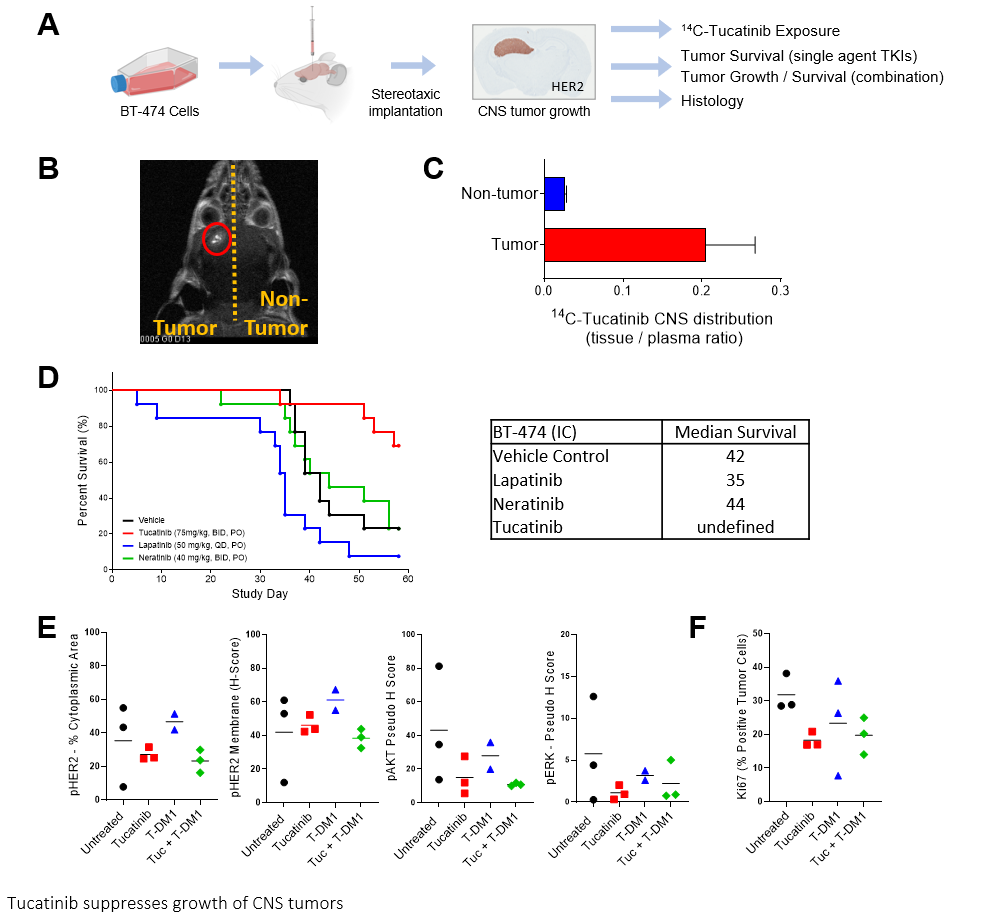
Supplementary Figure 6. Tucatinib suppresses growth of CNS tumors.

**A.** Schematic of intracranial xenograft model development and corresponding analysis. **B.** Image of mouse cranium with tumor-bearing region visible by contrast imaging. **C.** Analysis of ^14^C-tucatinib distribution in intracranial tumor-bearing and non–tumor-bearing tissue, expressed as a ratio of intracranial tissue radioactivity to plasma radioactivity. **D.** Kaplan–Meier survival plots of intracranially implanted BT-474 xenograft model, and table of median survival days post treatment with HER2-targeting TKIs. **E.** HALO-based quantification of histological analysis of phospho-epitopes downstream of HER2 signaling in intracranial BT-474-Redluc xenograft model. **F.** HALO-based quantification of histological analysis of Ki67 in intracranial BT-474-Redluc xenograft model.
